# Purification and pigment analysis of diadinoxanthin-binding PSI-LHCI supercomplexes from *Euglena gracilis* strain Z

**DOI:** 10.17912/micropub.biology.001678

**Published:** 2025-07-29

**Authors:** Runa Sakamoto, Ibuki Y. Takahashi, Koji Kato, Yoshiki Nakajima, Jian-Ren Shen, Ryo Nagao

**Affiliations:** 1 Research Institute for Interdisciplinary Science, Advanced Research Field, and Graduate School of Environmental, Life, Natural Science and Technology, Okayama University, Okayama 700-8530, Japan; 2 Faculty of Agriculture, Shizuoka University, Shizuoka 422-8529, Japan

## Abstract

*Euglena gracilis* exhibits a unique pigment profile distinct from land plants and green algae. In this study, we purified photosystem I supercomplexes containing light-harvesting complexes (PSI-LHCI) from *E. gracilis* strain Z and analyzed their biochemical and spectroscopic properties. The PSI-LHCI contained diadinoxanthin while lacking lutein and violaxanthin, which are characteristic of green-lineage organisms. The absorption and 77-K fluorescence spectra of *Euglena* PSI-LHCI showed the Qy peak of chlorophyll *a* at 675 nm and emission at 732 nm, respectively, comparable to land plant PSI-LHCI. These findings suggest conservation of long-wavelength chlorophylls despite distinct pigment-binding characteristics, shedding light on light-harvesting adaptations in secondary green algae.

**Figure 1. Purification and characterization of PSI-LHCI supercomplexes from E. gracilis strain Z:**
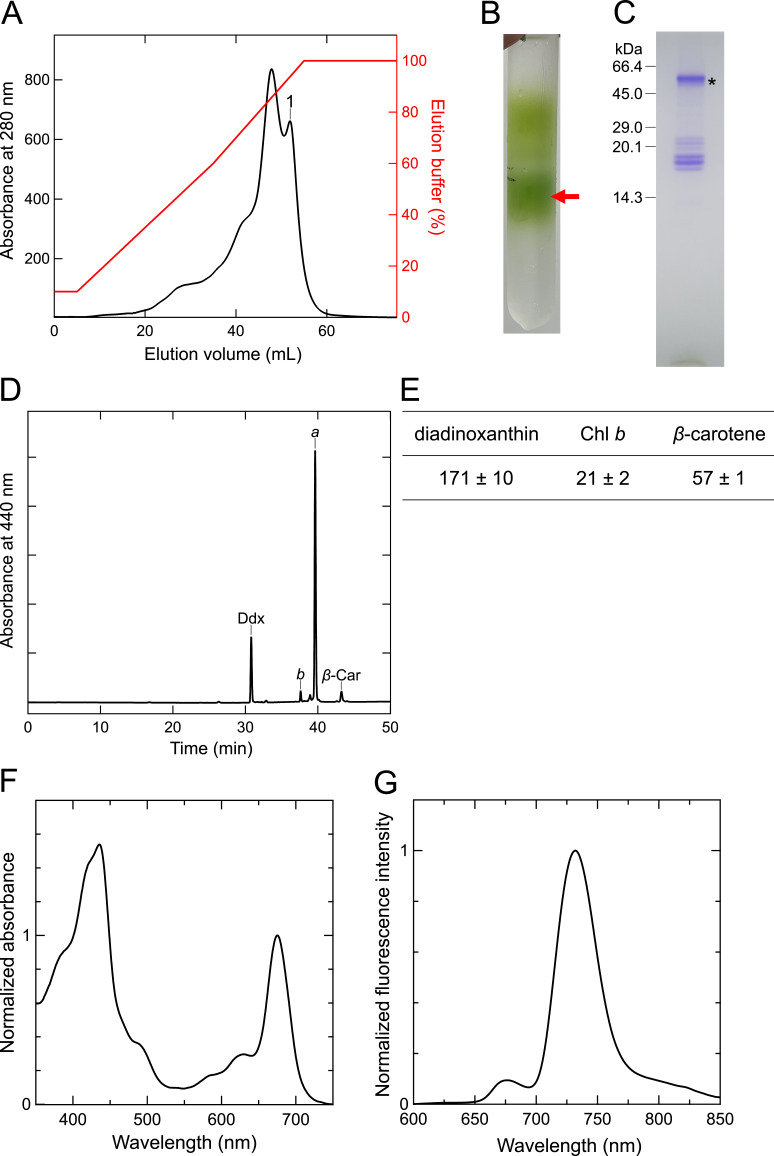
**(A)** Elution profile by anion exchange chromatography. The peak labeled as 1 was collected. ( **B)** Trehalose density gradient centrifugation profile of the peak 1. The green fraction (red arrow) was recovered as the *Euglena* PSI-LHCI supercomplex. **(C)** SDS-PAGE profile of the *Euglena* PSI-LHCI. The asterisk indicates a band assigned to PsaA and PsaB based on migration between 45.0 and 66.4 kDa molecular weight markers. **(D)** HPLC chromatogram of the *Euglena* PSI-LHCI monitored at 440 nm. Ddx, diadinoxanthin; *b* , Chl *b* ; *a* , Chl *a* ; *β* -Car, *β* -carotene. ( **E** ) Pigment composition of the *Euglena* PSI-LHCI. Values represent millimole of pigment per mole of Chl *a* and are shown as means ± S.D. of three independent measurements. **(F)** Absorption spectrum of the *Euglena* PSI-LHCI measured at room temperature, normalized to the Chl *a* Qy peak. **(G)** Fluorescence emission spectrum of the *Euglena* PSI-LHCI measured at 77 K upon excitation at 430 nm, normalized to the maximum intensity.

## Description

Photosystem I (PSI) catalyzes the light-driven electron transfer from plastocyanin or cytochrome *c*_6_ at the lumenal side of the thylakoid membrane to ferredoxin at the stromal side in oxygenic photosynthetic organisms (Golbeck 1992; Brettel and Leibl 2001; Fromme et al. 2001; Nelson and Junge 2015). In most species, PSI is associated with light-harvesting complexes (LHCs), forming PSI-LHCI supercomplexes, which facilitate excitation energy transfer. These pigment-protein assemblies vary considerably in both protein composition and pigment content across photosynthetic lineages (Croce and van Amerongen 2013, 2020; Hippler and Nelson 2021; Shen 2022).

*Euglena* *gracilis* , a phototrophic flagellate derived from secondary endosymbiosis (Turmel et al. 2009; Novák Vanclová et al. 2020), exhibits an unusual carotenoid (Car) profile. Its LHCs bind diadinoxanthin and diatoxanthin—Cars typically found in red-lineage organisms such as diatoms and haptophytes (Falkowski et al. 2004)—but lack lutein, violaxanthin, and zeaxanthin, which are characteristic of green-lineage organisms such as land plants and green algae (Cunningham Jr. and Schiff 1986; Casper-Lindley and Björkman 1998; Kato et al. 2017; Nagao et al. 2021). Despite this, the LHC polypeptides of *E. gracilis* display marked similarity to those of green algae (Houlné and Schantz 1988; Muchhal and Schwartzbach 1992), suggesting a conserved evolutionary origin. However, the PSI-LHCI supercomplex of *E. gracilis* has not been purified, and its molecular and functional properties remain unknown.

The PSI-LHCI supercomplexes of *E. gracilis* were purified using anion-exchange chromatography followed by trehalose density gradient centrifugation ( Figure 1A, B). The polypeptide composition of the purified complexes was analyzed by SDS-PAGE ( Figure 1C ). Multiple bands were detected, including a prominent band (asterisk) corresponding to PsaA and PsaB based on their migration between 45.0 and 66.4 kDa molecular weight markers. Additional bands within the 14.3–29.0 kDa range likely represent PSI and LHCI subunits. The overall band pattern closely resembled those previously reported for PSI-LHCI from land plants (Dunahay and Staehelin 1985; Croce et al. 1996) and green algae (Germano et al. 2002; Qin et al. 2015).

The pigment composition of *Euglena* PSI-LHCI was analyzed by HPLC ( Figure 1D ). Four pigments—diadinoxanthin, Chl *b* , Chl *a* , and *β* -carotene—were identified. The pigment stoichiometry relative to Chl *a* was calculated as 171 for diadinoxanthin, 21 for Chl *b* , and 57 for *β* -carotene ( Figure 1E ). Diadinoxanthin was the predominant Car, consistent with previous reports of isolated *Euglena* LHCs (Cunningham Jr. and Schiff 1986). This Car profile differed markedly from that of PSI-LHCI in land plants and green algae, whose LHCIs lack diadinoxanthin (Schmid et al. 2002; Klimmek et al. 2005; Qin et al. 2015; Shen 2022). Despite sequence similarity between *Euglena* and green algal LHC proteins (Houlné and Schantz 1988; Muchhal and Schwartzbach 1992), and their classification within the LHC protein superfamily (Engelken et al. 2010; Sturm et al. 2013), the distinct Car composition highlights unresolved mechanisms governing Car-binding specificity in *Euglena* .

The absorption spectrum of *Euglena* PSI-LHCI displayed the Qy band of Chl *a* at 675 nm, with additional peaks attributable to Chls and Cars in the 400–500 nm region ( Figure 1F ). Compared with PSI-LHCI from *Spinacia oleracea* (Qin et al. 2006) and *Chlamydomonas reinhardtii* (Drop et al. 2011), the *Euglena* complex exhibited lower absorbance in the 450–500 nm range, likely reflecting its distinct Car composition, particularly the absence of lutein and violaxanthin. Additionally, the Qy peak of Chl *a* in the *Euglena* PSI-LHCI appeared at a shorter wavelength (675 nm) than the corresponding peaks in *S. oleracea* and *C. reinhardtii* (679 nm), suggesting differences in Chl composition and binding properties.

The 77-K fluorescence-emission spectrum of *Euglena* PSI-LHCI exhibited a major peak at 732 nm with a broad shoulder near 677 nm ( Figure 1G ). The 732-nm emission closely resembles that of PSI-LHCI from land plants (Qin et al. 2006; Drop et al. 2011), but differs from the 714-nm peak characteristic of *C. reinhardtii* (Turmel et al. 2009; Le Quiniou et al. 2015) *.* In land plants, long-wavelength fluorescence near 730 nm originates from LHCI-bound Chls (Lam et al. 1984; Pålsson et al. 1995; Croce et al. 1998; Qin et al. 2006), suggesting that the 732-nm emission in *Euglena* similarly arises from LHCI-associated Chls. In contrast, the 677-nm shoulder likely reflects higher-energy Chls within LHCIs and/or dissociated LHCIs, consistent with the 675–680-nm fluorescence of isolated LHCIs from *S. oleracea* (Lam et al. 1984; Qin et al. 2006). Additionally, PSI-LHCI from other land plants exhibited emission peaks around 750 nm, attributed to specific Chl-binding sites within LHCIs (Li et al. 2024). This raises the possibility that similar sites contribute to the 732-nm emission in *Euglena* .

In summary, the presence of diadinoxanthin and the absence of canonical green-lineage Cars underscores the distinct pigment composition of *Euglena* PSI-LHCI, reflecting its divergence from typical land plant and green algae. Nevertheless, the 732-nm fluorescence emission indicates partial functional conservation with LHCI in land plants. Despite similarities in LHC protein sequences to green algae, the unique Car profile of *Euglena* suggests independent adaptations in pigment-binding properties. These observations expand our understanding of PSI-LHCI diversity and provide new insights into the structural and pigment-binding variations underlying photosynthetic light-harvesting systems.

## Methods

**Cell culture and thylakoid preparation**

*E. gracilis* strain Z was cultured in a Cramer-Myers medium (Cramer and Myers 1952) supplemented with 1/1000 volume of KW21 (Daiichi Seimo) at 30 °C under continuous aeration and a photosynthetic photon flux density of 30 µmol photons m ^−2^ s ^−1^ (Nagao et al. 2021). Harvested cells were pelleted by centrifugation and suspended in buffer A (20 mM Mes-NaOH (pH 6.5), 0.2 M trehalose, 5 mM CaCl _2_ , and 10 mM MgCl _2_ ). Cell disruption was performed using glass beads under dark and cold conditions on ice, employing 19 cycles of 10 s agitation and 3 min rest (Nagao et al. 2017). Unbroken cells were removed by centrifugation at 3,000 × g for 10 min at 4 °C. The resultant pellet was subjected to a second round of disruption under the same conditions, followed by centrifugation again at 3,000 × g for 10 min at 4 °C. The supernatant was then centrifuged at 125,000 × g for 20 min at 4 °C to isolate thylakoid membranes, which were resuspended in buffer A. Chl concentrations were determined in 100% methanol (Porra et al. 1989).

**Purification of PSI-LHCI supercomplexes**

All purification steps were performed at 4 °C unless otherwise stated. Thylakoid membranes were solubilized in the dark with 1% (w/v) sucrose monolaurate (SM; Carbosynth) at a Chl concentration of 0.25 mg mL ^−1^ by gentle stirring for 20 min on ice. After centrifugation at 100,000 × g for 20 min, the supernatant was applied to a HiTrap Q HP column (5 mL; Cytiva) equilibrated with buffer B (20 mM Mes-NaOH (pH 6.5), 0.2 M trehalose, and 0.03% SM). The column was washed with buffer B at a flow rate of 1.0 mL min ^−1^ until the eluate became colorless. Proteins were eluted using the following gradient: 0–5 min, 10% buffer C (buffer B with 500 mM NaCl); 5–35 min, 10–60% buffer C; 35–55 min, 60–100% buffer C; and 55–75 min, 100% buffer C. The peak labeled as 1 ( Figure 1A ) was collected and loaded onto a 10–40% linear trehalose gradient prepared in 20 mM Mes-NaOH (pH 6.5), 10 mM NaCl, and 0.03% SM. After centrifugation at 154,000 × g for 18 h (P40ST rotor; Hitachi), the green band (indicated by a red arrow in Figure 1B ) was harvested, concentrated using a 150 kDa cut-off filter (Apollo; Orbital Biosciences), and stored in liquid nitrogen.

**SDS-PAGE**

Protein composition was analyzed by SDS-PAGE as described by Ikeuchi and Inoue (1988). PSI-LHCI samples were solubilized in 3% lithium lauryl sulfate and 75 mM dithiothreitol at 60 °C for 10 min, and then applied to a 16% polyacrylamide gel containing 7.5 M urea. A molecular weight marker (SP-0110; APRO Science) was used. After electrophoresis, gels were stained with Coomassie Brilliant Blue R-250.

**Pigment analysis**

Pigments associated with PSI-LHCI were analyzed by HPLC as described previously (Nagao et al. 2020). Pigments were extracted in 100% methanol and separated using a flow rate of 0.9 mL min ^−1^ . The mobile phases consisted of solvent A (methanol:acetonitrile:0.25 M pyridine = 50:25:25 (v:v:v)) and solvent B (methanol:acetonitrile:acetone = 20:60:20 (v:v:v)) (Zapata et al. 2000). Pigments were identified by their absorption spectra and retention times (Zapata et al. 2000; Taniguchi and Lindsey 2021). Pigment composition was quantified according to the method of Nagao et al. (2021).

**Spectroscopic measurements**

Absorption spectra were measured at room temperature using a spectrophotometer (UV-2450; Shimadzu). Fluorescence-emission spectra were recorded at 77 K using a spectrofluorometer (RF-5300PC; Shimadzu). Each of the spectra was averaged and normalized.
